# Palliative psychological care in Germany: Results of a national online survey with psychologists and psycho-oncologists in specialist palliative care settings

**DOI:** 10.1177/26323524251406617

**Published:** 2026-01-13

**Authors:** Ricarda Scheiner, Isabel Sophie Burner-Fritsch, Martin Fegg, Berend Feddersen, Claudia Bausewein

**Affiliations:** 1 Department of Palliative Medicine, LMU University Hospital, Munich, Germany; 2 Psychotherapy Clinic, Prof. Dr. Fegg and Colleagues, Munich, Germany

**Keywords:** palliative psychology, psychologists, psycho-oncologists, seriously ill, death and dying, attitude, satisfaction, palliative care

## Abstract

**Background:**

Knowledge about the structure and type of palliative psychological care is limited and heterogeneous in most countries. Due to the lack of legal regulations in Germany, it is unclear how psychologists are integrated into multiprofessional palliative care teams (MPCT).

**Objectives:**

To describe how palliative psychology is organised in the various specialist palliative care settings for adults in Germany, how psychologists/psycho-oncologists assess their professional attitude and how satisfied they are with their work.

**Design:**

Online survey with psychologists/psycho-oncologists working in specialist palliative settings in Germany.

**Methods:**

The quantitative data was analysed descriptively and inferentially (correlations, group differences) using SPSS; category-based evaluation of free text comments.

**Results:**

One hundred and five of 210 respondents worked in a predominantly specialist palliative setting, mainly on palliative care units (137/210), mostly in combination with other settings (158/210), with a small proportion working in specialist palliative home care (29/210), palliative care support team within a hospital (28/210) or a hospice (16/210); the professional attitude did not differ between the predominantly and less palliative setting or the levels of professional experience. A high level of satisfaction with integration into and perceived appreciation by the MPCT, perceived effectiveness, supervision/intervision/exchange within the MPCT and overall job satisfaction was reported; when dissatisfaction was mentioned, the most common reasons given were a lack of resources, unfavourable structures or communication deficits; the predominantly palliative setting had a positive effect on satisfaction with integration into the MPCT, perceived effectiveness and overall satisfaction; a higher level of professional experience also showed a positive influence on perceived effectiveness.

**Conclusion:**

Although psychologists/psycho-oncologists were present in all palliative settings, they were not regularly a core member of the MPCT. Besides structural differences in palliative psychological care, there was also heterogeneity in the qualifications of the psychologists/psycho-oncologists. These results make the structure of psychological care in palliative care facilities in Germany more transparent and could be used to promote palliative psychology expertise as an integral part of multiprofessional care.

## Introduction

The multiprofessional approach in specialised palliative care aims to maintain and promote the quality of life of the seriously ill and their relatives.^1^ The European Association for Palliative Care recommends the integration of psychological expertise into the highly qualified treatment team to adequately recognise and alleviate distressing symptoms on a physical, psychological, social and spiritual level.^2^ The needs and concerns of people with advanced and life-limiting illness in a palliative situation, as well as the distress experienced by their loved ones, differ from classic psychotherapeutic or psycho-oncological perspectives.^3–5^

In Germany, palliative care is provided in palliative care units, hospital support teams, specialist palliative home care and inpatient hospices. Regardless of the form of care, the professional associations^2,6^ and the Council of Europe^7^ recommend multiprofessional and interdisciplinary cooperation between the various professional groups (medicine, nursing, psychology, social work, physiotherapy, artistic therapies, spiritual care, pharmacy, etc.). Even if the core team (minimum requirement) of doctors and nurses with specialised training should be supplemented by psychologists, social workers, physiotherapists and/or other professional groups, this has so far been regulated differently in Europe and is often a funding problem. Knowledge about the structure and content of palliative psychological care is limited and very heterogeneous in most countries.^8,9^ Foo et al.^8^ report that psychologists are underrepresented in palliative care, despite their valuable contribution to the care of the seriously ill and their relatives. This is due, among other things, to insufficient organisational integration, a lack of recognition and support, training and further education deficits, as well as unclear requirements and role profiles. In Germany, it is not yet known how palliative psychological support is provided in palliative settings.^9,10^ Some authors suggest involving social workers and nurses in palliative care more closely in the psychological support of those affected.^11–13^ In the United Kingdom, physicians and nurses are encouraged and trained to offer a certain level of psychological support in hospice care themselves, alongside psychological specialists and other therapeutic professionals.^14,15^ However, due to their extensive training, psychologists contribute competencies that cannot easily be taken over by other professional groups.^3,16–22^ Given the absence of legal regulation, it is unclear whether and with what kind of expertise psychologists are integrated into the multiprofessional team of specialised palliative care (MPCT), or whether psychologists are only irregularly consulted when needed.

Conventional psychotherapy by licensed psychotherapists is in Germany indicated if there is a ‘mental disorder with disease value’ and the curative mandate consists of ‘preventive and rehabilitative measures to promote health, which serve to establish, maintain, promote or regain mental and physical health [. . .]’.^23^ The focus in this setting is more on the aspect of restoring mental and physical performance by means of guideline-based diagnostics and manualised therapeutic approaches derived from this. Different circumstances, changes in the needs and symptoms of the dying, existential worries of relatives, and the usually very limited time and frequency of contacts place special demands on palliative psychologists.^18,22,24^ Psychologists should therefore develop and demonstrate an appropriate attitude and contribute specific knowledge, skills and competences to this professional field.^25,26^ Knowledge of the burdens and needs of the dying and their relatives, grief concepts, frequently encountered diseases, their course, treatment options and prognoses are indispensable.^3,24^ In addition to structural, ethical, legal, social or cultural expertise, Strada also emphasises the need for knowledge, skills, abilities in the areas of physical, psychological (and psychiatric), spiritual and existential aspects for comprehensive palliative care and a specifically supportive attitude for psychologists in this field.^3^ In Germany, cancer patients regularly receive psycho-oncological support in inpatient and outpatient care settings, wherever possible throughout the course of the disease.^27^ The basic qualification for acquiring the additional designation ‘psycho-oncologist’ is a university degree in medicine, psychology or other subjects such as social work or education. In practice, psycho-oncologists with differing degrees who have completed an additional specific psycho-oncological training certified by the German Cancer Society provide psycho-oncological support to patients. A nationwide survey and analysis of psycho-oncological care in Germany has shown that psycho-oncologists are also active in the various palliative care settings.^28^ While the focus of psycho-oncology is primarily on coping with psychological, physical and social problems in the course of cancer,^27^ palliative psychology aims explicitly at the needs and burden of patients with various life-limiting illnesses in the last phase of life and needs and concerns of their relatives.^16,18,22^ Even if there are commonalities and neither specialisation requires a licence to practise psychotherapy, palliative psychological support and interventions differ particularly in the immediate closeness to and intensive involvement with dying.^3,24^ Above all, the professional attitude and self-understanding of one’s own role seem crucial for building a sustainable and effective therapeutic alliance.^29,30^ For palliative psychologists in particular, Strada^3^ specifies an open, non-judgemental, appreciative, accepting, culturally sensitive, holistic, integrative and attentive attitude that enables the seriously ill, the dying and their relatives to open up and confide in their very individual suffering. To fulfil these requirements, a basic curriculum ‘Palliative Care for Psychologists’ was developed in Germany back in 2013.^31^ This qualification can be acquired at the Institute for Palliative Psychology^32^ and since 2020 it is possible to be certified as a specialist psychologist for palliative care.^33^ However, this training and certification is currently optional and not yet a requirement for working in this field. So far, 134 certificates have been issued.^34^ To provide high-quality support and care for people at the end of their lives, the literature emphasises not only a helpful professional attitude and specialist skills, but also the satisfaction of the psychologists themselves as an important aspect.^35,36^ Satisfaction with the job plays an important role in meeting the high demands of working with seriously ill and dying people and their relatives and preventing burnout.^35,37^ Compared to other areas of the medical care system, professionals in palliative settings do not appear to have a higher risk of developing burnout or compassion fatigue,^38,39^ but psychologists/psycho-oncologists have hardly been studied in this regard.^36^ Satisfaction in terms of team integration, perceived appreciation, perceived effectiveness, opportunities for exchange and overall satisfaction with work can have a protective effect on the development of burnout or compassion fatigue.^40,41^

Therefore, the aims of this study are:

(1) to investigate how palliative psychology is organised in the various palliative care settings for adults in Germany,
(2) which professional attitude characterises the psychologists/psycho-oncologists in their work with the seriously ill, the dying and their relatives,
(3) and how satisfied the psychologists/psycho-oncologists working in palliative care are with their integration into and the appreciation by the MPCT, the effectiveness of and their overall satisfaction with their work.

## Methods

### Study design

We conducted a web-based online survey among psychologists/psycho-oncologists in German palliative care settings for adults. The survey is reported following the Checklist for Reporting the Results of Internet e-Surveys (CHERRIES).^42^

### Development and pre-testing of the questionnaire

For this cross-sectional survey, an online questionnaire was developed that allows for different question formats (multiple choice with the option of multiple answers, open and closed questions, filter questions, rating questions). The entire survey contained 23 demographic questions, 45 questions on psychological interventions, 6 questions on professional attitude and 5 questions on satisfaction facets. In addition to literature research, the results of an online focus group discussion^43^ with six proven experts from the field of palliative psychology were used to construct the questionnaire. After a pre-test, the survey instrument was optimised and finally tested for feasibility and user-friendliness using a think-aloud protocol.^44^

### Recruitment process and description of the sample

**Figure 1.**
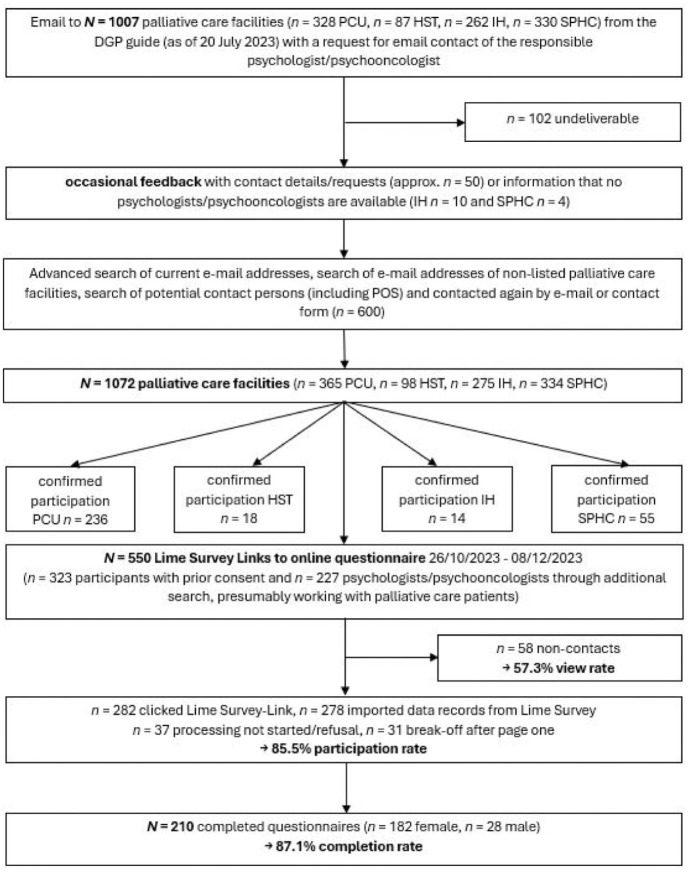
Flowchart of multi-stage sample recruitment and resulting sample reported according to CHERRIES guidelines.^42^ A total of *n* = 68 (24.5%) imported data sets were excluded due to missing data. DGP: Deutsche Gesellschaft für Palliativmedizin (German Association for Palliative Medicine); PCU: palliative care unit within a hospital; SPHC: specialist palliative home care; HST: palliative care support team within a hospital; IH: inpatient hospice; POS: psychological/psycho-oncological services or liaison services working in a consultative manner.

The target population for this study was psychologists/psycho-oncologists who regularly work with adult palliative care patients and their relatives throughout Germany. Palliative care units within hospitals (PCU), palliative care support teams within hospitals (HST), specialist palliative home care (SPHC), inpatient hospices (IH) and psychological/psycho-oncological services or liaison services (POS) working in a consultative manner were included as palliative settings. All identifiable palliative care facilities were contacted by email (*N* = 1072), informed about the study and asked to provide email contact of their responsible psychologists. The internet search has been extended to identify other potential psychologists working in palliative settings (see Figure 1). The newsletter of the German Association for Palliative Medicine (DGP) and an email to all members of the respective Section for Psychology informed them about the study and encouraged active participation.

### Survey administration

The questionnaire was realised and distributed using Lime Survey (a software platform for web-based surveys).^45^ To prevent multiple participation, only psychologists/psycho-oncologists who had received a personalised access link to the survey by email (*N* = 550) were able to complete the questionnaire. The invitation email contained brief information about the study and the Lime Survey link. The participants were informed about data protection conditions at the beginning of the anonymised survey and had to confirm that their participation was voluntary before processing. While completing the questionnaire, participants were able to scroll back or take a break. It was possible to terminate the questionnaire at any time without giving reasons. The data collection took place from the end of October until mid-December 2023. The survey was active for 6 weeks; after 4 weeks, a reminder email was sent via Lime Survey to those who had not yet started the survey.

### Description of the variables reported in this article

The selection of relevant variables and the construction of items relating to attitude and satisfaction were based on the results of a preliminary focus group discussion with recognised experts in the field of palliative psychology.

1. Personal details: Gender (simple selection), age (numerical free text), professional experience (PE) as a psychologist/psycho-oncologist in a palliative setting (numerical free text), federal state (free text).
2. Qualifications: Type of degree at Master’s/Diploma level (multiple choice), type of training and further education (multiple choice + free text field), type of licence to (psychotherapy) practise (multiple choice + free text field).
3. Working environment: Type of palliative setting (multiple choice + free text field), hours per week in palliative setting (multiple choice in numerical free text format), integration into MPCT (multiple choice + free text field), size of palliative setting operationalised as number of beds (PCU, HST, IH, hospital) or number of patients cared for (partial care) by SPHC (multiple selection in number/free text format), job allocation/job sharing (multiple selection and free text), additional supportive-therapeutic services in the palliative setting (multiple selection and free text).
4. Self-assessment professional attitude (7-point Likert scale from strongly disagree to agree fully and completely): Enter contact with a clear mission (presented inverted to prevent acquiescence), unintentional and open-ended attitude, attitude of not knowing and interested curiosity, presence for fear-free spaces, not only expertise but also intuition, proactively address wishes to hasten death (item A1 to A6).
5. Self-assessment satisfaction (7-point Likert scale from very bad to very good, cut-off at 2 = bad, then filter question with free text about the reason for the assessment): Satisfaction with integration into the MPCT, perceived effectiveness, perceived appreciation by the MPCT, satisfaction with supervision, intervision and exchange within the MPCT, overall job satisfaction (item S1 to S5).

### Data analysis

The returned data from the questionnaires were imported into the prepared data matrix (IBM SPSS Statistics – Version 29.0.2.0 [20]),^46^ processed and analysed. After transferring all processed questionnaires to the data matrix, the raw data was checked for missing values, influential data points, processing time, conspicuous time stamps and response patterns and adjusted according to predetermined exclusion criteria.

Sample characteristics are reported in mean values, standard deviations, median or percentages depending on their scaling level. Response behaviour on the attitude (A1 to A6) and satisfaction (S1 to S5) statements is presented in absolute numbers, percentage values, mean values, standard deviations, standard errors of the mean values, median, skewness, kurtosis and range. Intercorrelations between items of interest were calculated as Pearson or Spearman (Rho), depending on the scaling level.^47^ For analysing differences in response behaviour depending on actual activity in the palliative setting and professional experience, the variable ‘professional experience in the palliative setting’ was converted into a categorical one by means of percentile classification (two separating values, 33.33%: 0.05–3.0 years = PE low, 3.01–7.0 years = PE medium, 7.1–25.0 years = PE high) and a dichotomous variable was created to classify the activity in the palliative setting (‘predominantly palliative setting’ vs ‘less palliative setting’). Included in ‘predominantly palliative setting’ were those who indicated a predominant activity in PCU, SPHC, IH or HST. Since a large proportion of psychologists worked in POS, they were only included if the POS hours were less than PCU/HST/SPHC/IH or the hours were equally distributed (between POS and PCU/HST/SPHC/IH), as long as they were more than 10 h (e.g. 10 h/week in POS and 10 h/week in PCU/HST/SPHC/IH). Other settings were not included as these were private practice, bereavement care or counselling centres (from the free text information, it was not clear how much working time is spent on working with palliative care patients). Due to a lack of normal distribution on most of the dependent variables and hetero-scedasticity in some variables, differences in means for two independent samples were analysed using the Mann-Whitney *U* test (alpha level two-sided at 0.05) and for several independent samples using the Kruskal-Wallis *H* test (with Bonferroni correction for significance values).^47–49^ Cohen’s *d*, *η*^2^ or *r* were calculated as effect sizes.^50,51^ The MCAR (Missing Completely At Random) test according to Little^52,53^ showed that the pattern of the low proportion of missing values in the remaining data set (1.86%) depended on chance alone (*χ*^2^(5040) = 4800.818, *p* = 0.992). As no items were combined into scales, missing values were not imputed, and pairwise or listwise case exclusion was chosen for inferential statistical analyses.^54^

## Results

### Response rates and description of the sample

Of the *N* = 550 survey links sent, *n* = 58 were either undeliverable or non-functional (see Figure 1). After *n* = 278 data sets were imported into SPSS (*n* = 282 clicked Lime Survey link), *n* = 37 cases which did not start processing had to be removed and *n* = 31 cases were excluded because of premature break-off (possibly due to a technical problem or participants did not continue completing the questionnaire for other reasons) resulting in a view rate of 57.3% (282/492) and a participation rate of 85.5% (241/282). The completion rate of the final sample was 87.1% (210/241).

Most of the respondents were female (*n* = 182, 86.7%), with an average age of 44.77 years and professional experience in the palliative setting of 6.15 years (see Table 1). A large proportion of the respondents had studied psychology at a master’s degree (77.6%), and (cognitive) behavioural therapy was the most common type of licence training (37.6%) reported, whereby many participants (40.5%) did not have a licence as a psychological psychotherapist. Psycho-oncology (77.1%) was most frequently indicated as additional training/education, followed by further training in palliative care for psychologists (17.1%).

**Table 1.** Characteristics of the participating psychologists/psycho-oncologists.

Descriptive variable	*n*	*M* (SD)	Median (min–max)
Age^a^	209	44.77 (12.1)	43 (25–76)
Professional experience (palliative care) in years^a^	208	6.15 (4.9)	5 (0.5–25)
	*n* (%)	Further details	
Gender (*n*^a^ = 210)		*n* = 66 (31.4%) worked exclusively in a purely palliative setting (either only PCU, SPHC, HST or IH, or in a combination of these facilities)	
Female	182 (86.7%)		
Male	28 (13.3%)		
Master’s level degree (*n*^a^ = 204)^b^			
Psychology	163 (77.6%)	*n* = 9 (4.3%) with two master’s degrees	
Human medicine	20 (9.5%)		
Social pedagogy/social work	12 (5.5%)		
Other humanities or natural science degree programme	7 (3.3%)		
No master’s degree	2 (1%)		
License as a psychological psychotherapist (*n*^a^ = 202)^b^		*n* = 18 (8.6%) psychotherapy according to the ‘Heilpraktikergesetz’ *n* = 7 (4%) > one license as a psychological psychotherapist	
No licensed psychotherapist	85 (40.5%)		
(Cognitive) behavioural therapy	79 (37.6%)		
Depth psychology orientated/ psychodynamic therapy	27 (12.9%)		
Systemic therapy	7 (3.3%)		
Medical psychotherapy	5 (2.4%)		
Humanistic-oriented therapy	4 (1.9%)		
Psychotherapy for children and adolescents	3 (1.4%)		
Additional training and further education (*n*^a^ = 205)^b^		Number of additional training and further education programmes *n* = 72 (34.3%) one *n* = 49 (23.3%) two *n* = 33 (15.7%) three *n* = 22 (10.5%) four *n* = 6 (2.9%) five *n* = 6 (2.9%) > five	
Psycho-oncology	162 (77.1%)		
Palliative care for psychologists	36 (17.1%)		
Systemic counselling and therapy	33 (15.7%)		
Palliative care for psychosocial specialists	25 (11.9%)		
Hypnotherapy	22 (10.5%)		
Systemic counselling	17 (8.1%)		
Hypnosystemic therapy and counselling	6 (2.9%)		
Others	89 (42.4%)		
No training or further education	17 (8.1%)		

PCU: palliative care unit within a hospital; SPHC: specialist palliative homecare; HST: palliative care support team within a hospital; IH: inpatient hospice; POS: psychological/psycho-oncological services or liaison services working in a consultative manner.

a *N*’s range from 202 to 210 due to randomly missing data.

b *N* varies due to the possibility of multiple answers.

**Figure 2.**
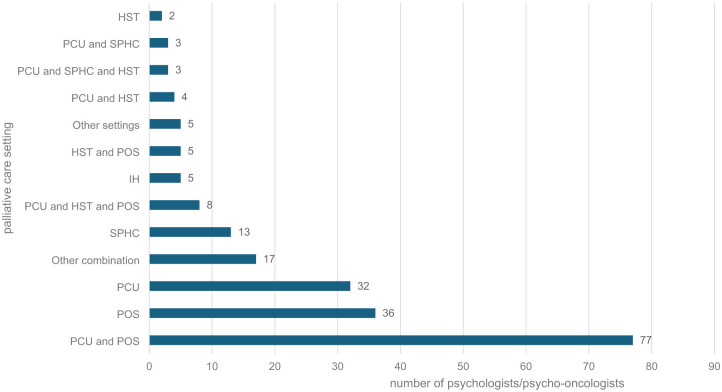
Distribution of psychologists/psycho-oncologists (*N* = 210) in the different (combinations of) palliative settings; *n* = 105 (50%) respondents worked in a predominantly palliative setting; *n* = 66 (31.4%) worked exclusively in a purely palliative setting (either only PCU, SPHC, HST or IH, or in a combination of these facilities). PCU: palliative care unit within a hospital; SPHC: specialist palliative homecare; HST: palliative care support team within a hospital; IH: inpatient hospice; POS: psychological/psycho-oncological services or liaison services working in a consultative manner.

**Figure 3.**
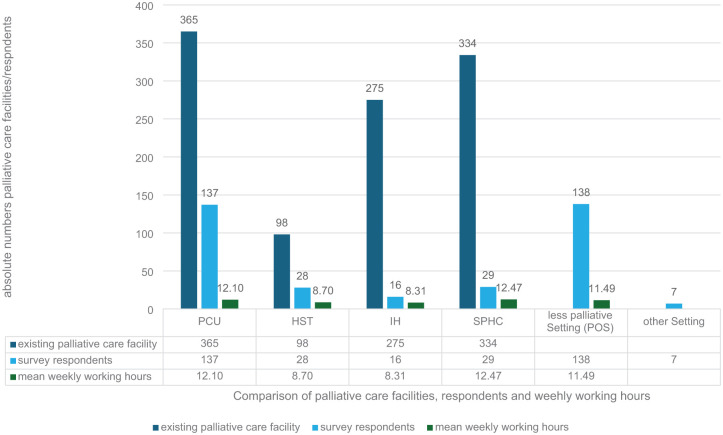
Comparison between the number of existing and contacted palliative care facilities in Germany (December 2023), the number of psychologists/psycho-oncologists who stated working a certain number of hours in the respective facilities and the mean weekly working hours. PCU: palliative care unit within a hospital; SPHC: specialist palliative homecare; HST: palliative care support team within a hospital; IH: inpatient hospice; POS: psychological/psycho-oncological services or liaison services working in a consultative manner.

The participants from all 16 federal states (see Table 2) were most frequently employed in a PCU and in the POS at the same time (36.7%), followed by employment in the POS (17.1%) or the PCU (15.2%) exclusively (see Figure 2). In total, there were 27 possible combinations of employment models, the most common of which are shown in Figure 2. Half of the total sample stated that they worked predominantly in a palliative setting, 31.4% (*n* = 66) exclusively in a palliative setting (either only PCU, SPHC, HST or IH, or in a combination of these facilities), and 57.1% stated that they were firmly integrated into an MPCT. The weekly working hours (see Figure 3) varied between the different settings, with the highest number of hours in SPHC (*M* = 12.47) and the lowest in the hospice (*M* = 8.31).

**Table 2.** Provenance of the participating psychologists/psycho-oncologists, integration into the multiprofessional team and further therapy programmes offered by the palliative care facilities.

Descriptive variable	*n*	Percent	Further details
Federal state (*n*^a^ = 210)			
Bavaria	47	22.4	
North Rhine-Westphalia	39	18.6	
Baden-Württemberg	25	11.9	
Rhineland-Palatinate	19	9	
Hessen	11	5.2	
Saxony	10	4.8	
Berlin	9	4.3	
Lower Saxony	9	4.3	
Hamburg	7	3.3	
Mecklenburg-Western Pomerania	7	3.3	
Saxony-Anhalt	7	3.3	
Schleswig-Holstein	6	2.9	
Thuringia	6	2.9	
Brandenburg	4	1.9	
Saarland	3	1.4	
Berlin-Brandenburg	1	0.5	
Integration into the multiprofessional team (*n*^a^ = 210)			Psychologists in charge for palliative patients *n* = 83 (39.5%) solely *n* = 50 (23.8%) two *n* = 38 (18.1%) three *n* = 41 (19.5%) more than three and depending on setting
Firmly integrated	120	57.1	
Consultative	56	26.7	
Firmly and consultative	23	11	
Other form of cooperation	11	5.2	
Additional therapy programmes (*n*^a^ = 208)^b^			
Pastoral care	194	92.4	Other programmes (free text) *n* = 15 (7.1%) physiotherapy/occupational therapy/logotherapy *n* = 25 (11.9%) complementary offers, for example, aromatherapy *n* = 9 (4.3%) volunteers *n* = 5 (2.4%) others
Music therapy	106	50.5	
Breathing therapy	102	48.6	
Art therapy	89	42.4	
Animal-assisted therapy	49	23.3	
Other therapy programmes	77	36.7	
None	4	1.9	

a *N*’s range from 208 to 210 due to randomly missing data.

b *N* varies due to the possibility of multiple answers.

### Professional attitude and satisfaction facets

Table 3 shows the exact wording and the response behaviour of the participants on the six items regarding the professional attitude (A1–A6) and the satisfaction facets (S1–S5) in absolute numbers and percentages. All items except for A1 (= reversed item in which the negation rather than the affirmation is symptomatic of a high level of characteristic expression^55^) are left skewed and not normally distributed. The low discriminatory power of most items is also evident from the fact that only 3 of the 11 items (A1, A3, S4) utilised all seven response categories. For one item (A4) only four of the seven response categories were used. The mean values, standard deviations, medians, skewness and kurtosis of the self-assessments (see Table 4) provide a more detailed analysis of the strong agreement tendency on most items. Psychologists/psycho-oncologists agreed less with the statement A1 (after recoding) than with the other items. Agreement with items A2 to A6 was substantially high. The descriptive statistics of the items S1 to S5 show a very uniform picture, characterised by predominant satisfaction, effectiveness and perceived appreciation.

**Table 3.** Response behaviour to the items palliative psychological attitude and satisfaction among psychologists/psycho-oncologists (*n* = 210).

Item attitude *A1* to *A6*	*n* ^a^	Proportion of agreement *n* (valid %)						
		Strongly disagree (1)	Disagree (2)	Tend to disagree (3)	Partially/partially (4)	Rather agree (5)	Agree (6)	Agree fully and completely (7)
*A1.* In my work with the seriously ill, the dying, their relatives and loved ones, I consider it important to start with a *clear mission* at the first contact^b^	205	15 (7.3%)	57 (27.8%)	53 (25.9%)	46 (22.4%)	19 (9.3%)	6 (2.9%)	9 (4.4%)
*A2.* In my work with the seriously ill, the dying, their relatives and loved ones, I consider it important to approach people with an *open-minded and open-ended* attitude	205	1 (0.5%)	–	4 (2%)	10 (4.9%)	16 (7.8%)	72 (35.1%)	102 (49.8%)
*A3.* In my work with the seriously ill, the dying, their relatives and loved ones, I consider it important to approach people with an attitude of *not knowing and interested curiosity*	204	2 (1%)	8 (3.9%)	9 (4.4%)	29 (14.2%)	31 (15.2%)	61 (29.9%)	64 (31.4%)
*A4.* In my work with the seriously ill, the dying, their relatives and loved ones, I consider it important to open up *fear-free spaces* through my attitude and *presence*, which enables the unspeakable to be expressed	204	–	–	–	2 (1%)	4 (2%)	46 (22.5)	152 (74.5%)
*A5.* In my work with the seriously ill, the dying, their relatives and loved ones, I consider it important *not only* to use my *professional expertise, but also* to follow my *intuition*, for example, when it comes to deciding which topics should perhaps be left ‘outside’ during counselling	205	–	1 (0.5%)	3 (1.5%)	8 (3.9%)	21 (10.2%)	80 (39%)	92 (44.9%)
*A6.* In my work with the seriously ill, the dying, their relatives and loved ones, I consider it important to *proactively address* the existence of *wishes to hasten death*	204	–	3 (1.5%)	11 (5.4%)	33 (16.2%)	39 (19.1%)	69 (33.8%)	49 (24%)
Item satisfaction *S1* to *S5*	*n* ^a^	Proportion of agreement *n* (valid %)						
		Very bad (1)	Bad (2)	Rather bad (3)	Partially/partially (4)	Satisfactory (5)	Good (6)	Very good (7)
*S1.* How well do you feel *integrated into the multiprofessional team* (of the palliative care ward/SPHC/IH or palliative care service)? (Here we would like to know to what extent you perceive yourself as part of the multiprofessional team.)	201	–	1 (0.5%)	14 (7%)	21 (10.4%)	22 (10.9%)	64 (31.8%)	79 (39.3%)
*S2.* How do you evaluate the *effectiveness of your work*? (e.g. based on positive, negative or no feedback from patients and/or their relatives or from the team. Or also based on your own assessment of effectiveness based on perceived/not or barely perceptible changes in patients and/or their relatives.)	204	–	–	1 (0.5%)	13 (6.4%)	29 (14.2%)	119 (58.3%)	42 (20.6%)
*S3.* How do you experience the *appreciation of your work* by the multiprofessional team? (You notice this, e.g. in the recognition expressed, the utilisation of and attention paid to your expertise, or the importance attached to your work.)	202	–	1 (0.5%)	3 (1.5%)	21 (10.4%)	22 (10.9%)	90 (44.6%)	65 (32.2%)
*S4.* How do you evaluate the existing opportunities for *supervision, intervision or exchange within the multiprofessional* team for your own mental healthcare?	201	4 (2%)	8 (4%)	7 (3.5%)	24 (11.9%)	42 (20.9%)	81 (40.3%)	35 (17.4%)
*S5.* All in all, *how satisfied would you rate your work* in the palliative setting?	204	–	–	5 (2.5%)	11 (5.4%)	21 (10.3%)	108 (52.9%)	59 (28.9%)

a The varying *n*’s are due to randomly missing values. Assessment of the items on 7-point Likert scale from strongly disagree to agree fully and completely for the items ‘attitude’ and from very bad to very good for the items ‘satisfaction’.

b Inverted item was reversed for further calculation.

**Table 4.** Mean values, standard deviations, standard errors of the mean values, median, skewness, kurtosis and range of the analysed items.

Item	*n* ^a^	*M*	*SD*	*SE*	*Median*	Skewness	Kurtosis	Range
*A1.* Attitude ‘enter contact with a clear mission’	205	4.75	1.4	0.101	5.0	−0.725	0.271	6
*A2.* Attitude ‘unintentional and open-ended’	205	6.24	1.01	0.070	6.0	−1.829	4.373	6
*A3.* Attitude ‘not knowing and interested curiosity’	204	5.54	1.45	0.102	6.0	−0.970	0.329	6
*A4.* Attitude ‘presence for fear-free spaces’	204	6.71	0.55	0.039	7.0	−2.098	5.218	3
*A5.* Attitude ‘not only expertise but also intuition’	205	6.20	0.94	0.065	6.0	−1.501	2.826	5
*A6.* Attitude ‘proactively address wishes to hasten death’	204	5.50	1.25	0.087	6.0	−0.631	−0.326	5
*S1.* Satisfaction ‘with integration into the team’	201	5.85	1.27	0.090	6.0	−0.996	−0.004	5
*S2.* Satisfaction ‘perceived effectiveness’	204	5.92	0.80	0.056	6.0	−0.839	0.965	4
*S3.* Satisfaction ‘perceived appreciation by the team’	202	5.94	1.03	0.072	6.0	−1.066	0.880	5
*S4.* Satisfaction ‘with supervision, intervision and exchange within the team’	201	5.36	1.38	0.098	6.0	−1.179	1.237	6
*S5.* Satisfaction ‘overall job satisfaction’	204	6.0	0.91	0.064	6.0	−1.228	1.821	4

a The varying *n*’s are due to randomly missing values. Assessment of the items on 7-point Likert scale from strongly disagree to agree fully and completely for the items ‘attitude’ and from very bad to very good for the items ‘satisfaction’.

### Relationships between variables of interest

The intercorrelations (see Table 5) between the items A1–A6, S1–S5 and the two metric variables age and PE show a few possible relationships. Age and PE correlate highly with each other (*r* = 0.51, *p* < 0.001), and a higher age also shows a stronger agreement on the items A5 (*r* = 0.20, *p* = 0.004), S2 (*r* = 0.32, *p* < 0.001) and S5 (*r* = 0.16, *p* = 0.023). A higher level of PE is associated with stronger agreement on items S1 (*r* = 0.17, *p* = 0.017), S2 (*r* = 0.26, *p* < 0.001) and S3 (*r* = 0.16, *p* = 0.024). The intercorrelations between items A2 and A6 show positive and in some cases strong correlations, the strongest relationship is found between A2 and A3 (*r* = 0.45, *p* < 0.000). Few positive correlations are found for item A1 (only with A2). It is negatively correlated with A3, A4, A5 and A6. The correlations between the satisfaction measures S1–S5 are also consistently strong and point in a positive direction, with the strongest relationship between S1 und S3 (*r* = 0.63, *p* < 0.000).

**Table 5.** Intercorrelations of the metric (Pearson) and interval-scaled (Spearman-Rho) variables (pairwise case exclusion).

Item	1	2	3 A1	4 A2	5 A3	6 A4	7 A5	8 A6	9 S1	10 S2	11 S3	12 S4	13 S5
1. Age	–												
2. Years professional experience	0.51**	–											
3. *A1.* Attitude ‘enter contact with a clear mission’	0.06	0.08	–										
4. *A2.* Attitude ‘unintentional and open-ended’	0.14	0.09	0.14*	–									
5. *A3.* Attitude ‘not knowing and interested curiosity’	0.06	−0.03	−0.01	0.45**	–								
6. *A4.* Attitude ‘presence for fear-free spaces’	−0.10	−0.11	−0.02	0.27**	0.34**	–							
7. *A5.* Attitude ‘not only expertise but also intuition’	0.20**	0.11	−0.03	0.19**	0.26**	0.16*	–						
8. *A6.* Attitude ‘proactively address wishes to hasten death’	−0.05	0.04	−0.04	0.08	0.08	0.16*	0.12	–					
9. *S1.* Satisfaction ‘with integration into the team’	0.09	0.17*	0.14*	0.14*	−0.01	0.12	0.08	0.18*	–				
10. *S2.* Satisfaction ‘perceived effectiveness’	0.32**	0.26**	0.06	0.10	−0.02	0.12	0.12	−0.03	0.39**	–			
11. *S3.* Satisfaction ‘perceived appreciation by the team’	0.06	0.16*	0.08	0.09	−0.00	0.14*	0.06	0.18*	0.63**	0.52**	–		
12. *S4.* Satisfaction ‘with supervision, intervision and exchange within the team’	−0.04	0.10	−0.00	0.03	−0.06	0.04	−0.02	0.15*	0.46**	0.25**	0.45**	–	
13. *S5.* Satisfaction ‘overall job satisfaction’	0.16*	0.11	0.13	0.12	0.06	0.12	0.03	0.05	0.53**	0.54**	0.54**	0.40**	–

* The correlation is significant at the 0.05 level (two-sided).

** The correlation is significant at the level of 0.01 (two-sided).

### Differences in terms of palliative setting and professional experience

The Mann-Whitney *U* tests show that the agreement on items A1 to A6 do not differ significantly between participants who work in a predominantly palliative setting and those in a less palliative setting (see Table 6). The group differences on items S1–S5 are more heterogeneous (see Table 7). The statements relating to S1 (*U*(103, 98) = 6524.5, *p* < 0.001, *d*_Cohen_ = 0.52), S2 (*U*(103, 101) = 6028.0, *p* = 0.027, *d*_Cohen_ = 0.23) and S5 (*U*(103, 101) = 6339.5, *p* = 0.003, *d*_Cohen_ = 0.39) differ significantly, albeit only with small to medium effect sizes. Participants who work predominantly in a palliative setting thus seem to feel better integrated into the MPCT, rate the effectiveness of their work as higher and report greater overall satisfaction. There are no significant differences for items S3 and S4.

**Table 6.** Differences in mean values for attitude measures (A1–A6) depending on group affiliation ‘predominantly palliative setting’ (*N* = 210).

Attitude measures	Predominantly palliative setting (*n* = 105)				Less palliative setting (*n* = 105)				Mann-Whitney *U* test					
	*n* ^c^	*M*	SD	Median	*n* ^c^	*M*	SD	Median	*z*	*U*	*d*_Cohen_	*η* ^2^	*r* ^b^	*p* ^a^
*A1.* Attitude ‘enter contact with a clear mission’	103	4.86	1.34	5	102	4.64	1.54	5	1.075	5697.5	0.15	0.005	0.08	0.283
*A2.* Attitude ‘unintentional and open-ended’	103	6.24	0.98	7	102	6.24	1.05	6	0.004	5254.5	0	0	0	0.997
*A3.* Attitude ‘not knowing and interested curiosity’	103	5.52	1.45	6	101	5.55	1.46	6	−0.201	5119.5	0.03	0	0.01	0.841
*A4.* Attitude ‘presence for fear-free spaces’	103	6.70	0.52	7	101	6.71	0.59	7	−0.529	5032.5	0.06	0.001	0.04	0.597
*A5.* Attitude ‘not only expertise but also intuition’	103	6.24	0.87	6	102	6.17	1.01	6	0.234	5344.5	0.03	0	0.02	0.815
*A6.* Attitude ‘proactively address wishes to hasten death’	103	5.55	1.19	6	101	5.46	1.31	6	0.389	5360.0	0.05	0.001	0.03	0.698

The non-parametric Mann-Whitney *U* test compares independent samples (of approximately equal size) for variables that are not normally distributed.^49^ The alpha level tested (two-sided) is 5%.

a Asymptotic significance.

b The effect size *r* was calculated using the following equation: $r=\frac{z}{\sqrt{N}}$ . The effect sizes are interpreted according to Cohen^51^: *r* = 0.10–0.20/*d*_Cohen_ = 0.20–0.40/*η*^2^ = 0.010–0.039 (small effect), *r* = 0.24–0.33/*d*_Cohen_ = 0.50–0.70/*η*^2^ = 0.060–0.110 (intermediate effect), *r* = 0.37–0.45/*d*_Cohen_ = 0.80 to ⩾1.0/*η*^2^ = 0.140–0.200 (large effect), *r* ⩾ 0.50 (strong effect).

c The varying *n*’s are due to randomly missing values. *d*_Cohen_ and *η*^2^ were calculated using the effect size calculator from psychometrica.^50^

**Table 7.** Differences in mean values for satisfaction measures (S1–S5) depending on group affiliation ‘predominantly palliative setting’ (*N* = 210).

Satisfaction measures	Predominantly palliative setting (*n* = 105)				Less palliative setting (*n* = 105)				Mann-Whitney *U* test					
	*n* ^c^	*M*	SD	Median	*n* ^c^	*M*	SD	Median	*z*	*U*	*d*_Cohen_	*η* ^2^	*r* ^b^	*p* ^a^
*S1.* Satisfaction ‘with integration into the team’	103	6.18	1.06	6	98	5.49	1.38	6	3.769	6524.5	0.52	0.064	0.27	**<0.001**
*S2.* Satisfaction ‘perceived effectiveness’	103	6.03	0.81	6	101	5.81	0.78	6	2.206	6028.0	0.23	0.019	0.15	0.027
*S3.* Satisfaction ‘perceived appreciation by the team’	103	5.96	1.08	6	99	5.92	0.98	6	0.754	5391.5	0.10	0.002	0.05	0.451
*S4.* Satisfaction ‘with supervision, intervision and exchange within the team’	103	5.44	1.33	6	98	5.29	1.44	6	0.656	5306.0	0.10	0.002	0.05	0.512
*S5.* Satisfaction ‘overall job satisfaction’	103	6.17	0.88	6	101	5.84	0.92	6	2.970	6339.5	0.39	0.036	0.21	0.003

The non-parametric Mann-Whitney *U* test compares independent samples (of approximately equal size) for variables that are not normally distributed.^49^ The alpha level tested (two-sided) is 5%.

a Asymptotic significance.

b The effect size *r* was calculated using the following equation: $r=\frac{z}{\sqrt{N}}.$ The effect sizes are interpreted according to Cohen^51^: *r* = 0.10–0.20/*d*_Cohen_ = 0.20–0.40/*η*^2^ = 0.010–0.039 (small effect), *r* = 0.24–0.33/*d*_Cohen_ = 0.50–0.70/*η*^2^ = 0.060–0.110 (intermediate effect), *r* = 0.37–0.45/*d*_Cohen_ = 0.80 to ⩾1.0/*η*^2^ = 0.140–0.200 (large effect), *r* ⩾ 0.50 (strong effect).

c The varying *n*’s are due to randomly missing values. *d*_Cohen_ and *η*^2^ were calculated using the effect size calculator from psychometrica.^50^

Kruskal-Wallis *H* tests reveal no significant differences between participants with low, medium and high levels of PE regarding items A1 to A6 (see Table 8). Regarding the satisfaction measures, those with more PE differ from those with less PE only in the variable S2 (*χ*^2^(2, *N* = 203) = 10.325, *p* = 0.006, *d*_Cohen_ = 0.42) significantly (see Table 9). Post hoc comparisons using the Dunn method with Bonferroni correction for multiple testing show that participants with less PE experience themselves less effectively (*M* = 5.68) than those with medium (*M* = 6.03, *p* = 0.008/ adjusted *p* = 0.025) or high (*M* = 6.09, *p* = 0.005/ adjusted *p* = 0.014) PE. There is no significant difference between those with medium and high PE. There are no significant differences in agreement on the variables S1, S3, S4 and S5. The free text comments from participants (42/210) who rated the satisfaction items as bad to very bad are summarised in Table 10 on a category basis.

**Table 8.** Differences in mean values for attitude measures depending on group affiliation ‘professional experience’.

		*A1.* Attitude ‘enter contact with a clear mission’	*A2.* Attitude ‘unintentional and open-ended’	*A3.* Attitude ‘not knowing and interested curiosity’	*A4.* Attitude ‘presence for fear-free spaces’	*A5.* Attitude ‘not only expertise but also intuition’	*A6.* Attitude ‘proactively address wishes to hasten death’
Summary of the Kruskal-Wallis test for independent samples		*N*^a^ = 203 *H*(2) = 1.519 *η*^2^ = 0.002 *d*_Cohen_ = 0.10 p* = 0.468	*N*^a^ = 203 *H*(2) = 3.818 *η*^2^ = 0.009 *d*_Cohen_ = 0.19 p* = 0.148	*N*^a^ = 202 *H*(2) = 1.863 *η*^2^ = 0.001 *d*_Cohen_ = 0.05 p* = .394	*N*^a^ = 202 *H*(2) = 1.366 *η*^2^ = 0.003 *d*_Cohen_ = 0.11 p* = 0.505	*N*^a^ = 203 *H*(2) = 2.399 *η*^2^ = 0.002 *d*_Cohen_ = 0.09 p* = 0.301	*N*^a^ = 202 *H*(2) = 0.828 *η*^2^ = 0.006 *d*_Cohen_ = 0.15 p* = 0.661
*PE low* 0.50–3.00 years	*n*	78	78	78	78	78	77
	*M* (SD)	4.62 (1.46)	6.05 (1.15)	5.5 (1.47)	6.73 (0.57)	6.04 (1.06)	5.51 (1.33)
	Mdn	5	6	6	7	6	6
*PE medium* 3.01–7.00 years	*n*	60	60	60	60	60	60
	*M* (SD)	4.75 (1.60)	6.33 (0.91)	5.73 (1.33)	6.72 (0.52)	6.32 (0.79)	5.62 (1.17)
	Mdn	5	7	6	7	6	6
*PE high* 7.00–25.0 years	*n*	65	65	64	64	65	65
	*M* (SD)	4.94 (1.29)	6.35 (0.90)	5.36 (1.54)	6.66 (0.58)	6.28 (0.90)	5.42 (1.24)
	Mdn	5	7	6	7	6	6
Pairwise sample comparison							
Sample 1 with sample 2		*PE low – PE medium z* = −0.723, p* = .469, *p*^$^ = 1.000 *PE low – PE high z* = −1.215, p* = 0.224, *p*^$^ = 0.673 *PE medium – PE high z* = −0.446, p* = 0.655, *p*^$^ = 1.000	*PE low – PE medium z* = −1.600, p* = .110, *p*^$^ = .329 *PE low – PE high z* = −1.716, p* = .086, *p*^$^ = .258 *PE medium – PE high z* = −0.076, p* = 0.940, *p*^$^ = 1.000	*PE high – PE low z* = 0.522, p* = 0.602, *p*^$^ = 1.000 *PE high – PE medium z* = 1.350, p* = 0.177, *p*^$^ = 0.531 *PE low – PE medium z* = −0.900, p* = 0.368, *p*^$^ = 1.000	*PE high – PE medium z* = 0.717, p* = 0.473, *p*^$^ = 1.000 *PE high – PE low z* = 1.160, p* = 0.246, *p*^$^ = 0.738 *PE medium – PE low z* = 0.389, p* = 0.697, *p*^$^ = 1.000	*PE low – PE high z* = −1.250, p* = 0.211, *p*^$^ = 0.633 *PE low – PE medium z* = −1.375, p* = 0.169, *p*^$^ = 0.508 *PE high – PE medium z* = 0.146, p* = 0.884, *p*^$^ = 1.000	*PE high – PE low z* = .577, p* = 0.564, *p*^$^ = 1.000 *PE high – PE medium z* = 0.899, p* = 0.368, *p*^$^ = 1.000 *PE low – PE medium z* = −0.371, p* = 0.711, *p*^$^ = 1.000

PE: professional experience.

The metric variable was categorised by percentile classification with two separating values at 33.33% (*N* = 208). The non-parametric Kruskal-Wallis test compares means of more than two independent samples for variables that are not normally distributed. It uses ranks instead of the actual values.

a The varying *n*’s are due to randomly missing values. *d*_Cohen_ and *η*^2^ were calculated using the effect size calculator from psychometrica.^50^

* Asymptotic significances (two-sided tests) with significance level at 0.050.

$ Significance values are adjusted by the Bonferroni correction for several tests. The effect sizes are interpreted according to Cohen^51^: *d*_Cohen_ = 0.20–0.40/*η*^2^ = 0.010–0.039 (small effect), *d*_Cohen_ = 0.50–0.70/*η*^2^ = 0.060–0.110 (intermediate effect), *d*_Cohen_ = 0.80 to ⩾1.0/*η*^2^ = 0.140–0.200 (large effect).

**Table 9.** Differences in mean values for satisfaction measures depending on group affiliation ‘professional experience’.

		*S1.* Satisfaction ‘with integration into the team’	*S2.* Satisfaction ‘perceived effectiveness’	*S3.* Satisfaction ‘perceived appreciation by the team’	*S4.* Satisfaction ‘with supervision, intervision and exchange within the team’	*S5.* Satisfaction ‘overall job satisfaction’
Summary of the Kruskal-Wallis test for independent samples		*N*^a^ = 199 *H*(2) = 5.291 *ƞ*^2^ = 0.017 *d*_Cohen_ = 0.26 p* = 0.071	*N*^a^ = 203 *H*(2) = 10.325 ***ƞ***^2^ = 0.**042** ***d*_Cohen_** = **0.42** ***p*** * = 0.**006**	*N*^a^ = 201 *H*(2) = 3.185 *ƞ*^2^ = .006 *d*_Cohen_ = 0.16 p* = 0.203	*N*^a^ = 200 *H*(2) = 5.650 *ƞ*^2^ = 0.019 *d*_Cohen_ = 0.28 p* = 0.059	*N*^a^ = 203 *H*(2) = 4.573 *ƞ*^2^ = 0.013 *d*_Cohen_ = 0.23 p* = 0.102
*PE low* 0.50–3.00 years	*n*	77	77	77	77	77
	*M* (SD)	5.53 (1.47)	5.68 (0.91)	5.73 (1.20)	5.09 (1.52)	5.83 (1.04)
	Mdn	6	6	6	6	6
*PE medium* 3.01–7.00 years	*n*	60	60	59	60	60
	*M* (SD)	6.1 (1.05)	6.03 (0.80)	6.05 (0.96)	5.67 (1.20)	6.18 (0.81)
	Mdn	6	6	6	6	6
*PE high* 7.00–25.0 years	*n*	62	66	65	63	66
	*M* (SD)	6.03 (1.12)	6.09 (0.58)	6.09 (0.84)	5.41 (1.34)	6.08 (0.77)
	Mdn	6	6	6	6	6
Pairwise sample comparison							
Sample 1 with sample 2		*PE low – PE high z* = −1.811, p* = 0.070, *p*^$^ = 0.210 *PE low – PE medium z* = −2.072, ***p*** * = **0.038**, *p*^$^ = 0.115 *PE high – PE medium z* = 0.264, p* = 0.792, *p*^$^ = 1.000	*PE low – PE medium z* = −2.632, ***p*** * = **0.008**, ***p***^$^ = **0.025** *PE low – PE high z* = −2.830, ***p*** * = **0.005**, ***p***^$^ = **0.014** *PE medium – PE high z* = −0.120, p* = 0.905, *p*^$^ = 1.000	*PE low – PE medium z* = −1.485, p* = 0.137, *p*^$^ = 0.412 *PE low – PE high z* = −1.548, p* = 0.122, *p*^$^ = 0.365 *PE medium – PE high z* = −0.021, p* = .983, *p*^$^ = 1.000	*PE low – PE high z* = −1.257, p* = 0.209, *p*^$^ = 0.626 *PE low – PE medium z* = −2.366, ***p*** * = **0.018**, *p*^$^ = 0.054 *PE high – PE medium z* = 1.075, p* = 0.282, *p*^$^ = 0.847	*PE low – PE high z* = −1.110, p* = 0.267, *p*^$^ = 0.801 *PE low – PE medium z* = −2.132, ***p*** * = **0.033**, *p*^$^ = 0.099 *PE high – PE medium z* = 1.014, p* = 0.311, *p*^$^ = 0.932

PE: professional experience.

The metric variable was categorised by percentile classification with two separating values at 33.33% (*N* = 208). The non-parametric Kruskal-Wallis test compares means of more than two independent samples for variables that are not normally distributed. It uses ranks instead of the actual values.

a The varying *n*’s are due to randomly missing values. *d*_Cohen_ and *η*^2^ were calculated using the effect size calculator from psychometrica.^50^

* Asymptotic significances (two-sided tests) with significance level at 0.050.

$ Significance values are adjusted by the Bonferroni correction for several tests. The effect sizes are interpreted according to Cohen^51^: *d*_Cohen_ = 0.20–0.40/*η*^2^ = 0.010–0.039 (small effect), *d*_Cohen_ = 0.50–0.70/*η*^2^ = 0.060–0.110 (intermediate effect), *d*_Cohen_ = 0.80 to ≥1.0/*η*^2^ = 0.140–0.200 (large effect).

**Table 10.** Category-based analysis of free text comments from those respondents who rated their satisfaction on the items S1 to S5 as bad to very bad and gave a reason for this.

Content category	Examples from free text
S1 satisfaction ‘with integration into team’ bad to very bad (14/201)	
Lack of time/personnel resources (*n* = 9)	‘*Finally, there is simply not enough time for the patients*’
Unfavourable clinic structures, such as too few posts for palliative patients or too many responsibilities for different areas (*n* = 7)	‘*I work in the consultative manner. [. . .] there is a lack of time for regular dialogue*’. ‘*Irregular participation in patient meetings, due to the fact that I am responsible for three teams and only have limited time resources*’ ‘*Clinic structures*’
Lack of dialogue within the multiprofessional team (*n* = 6)	‘*An exchange about the patients only takes place once a week. The multiprofessional team could benefit much more from each other. [. . .] If we are requested for a consultation, this is usually done without any information regarding the burden or type or “current status” of the illness: the important thing is that the consultation is processed, nobody is interested in feedback regarding our work*’
Lack of knowledge or interest in the role of palliative psychology on the part of the team (*n* = 4)	‘*In addition, the doctors decide which patients are offered psychological support. This means that patients and relatives fall through the cracks*’
Lack of integration by the responsible leader (*n* = 4)	‘*Spatial separation of my office from the offices of the other team members*’ ‘*No psychological support is foreseen for the palliative care unit on which I look after patients; I am only consulted sporadically by the attending physician*’
Existing tensions/conflicts within the multiprofessional team (*n* = 2)	‘*Openness of the team, tensions within the team (within and between different professional groups), lack of staff, little room for communication, little knowledge of psychology and psycho-oncology (What is the role of POS? Where are the boundaries? What “can” POS do? Who can do POS?), no common language*’
S2 satisfaction ‘with perceived effectiveness’ bad to very bad (1/204)	
Lack of time/personnel resources (*n* = 1)	‘*Too few resources for several conversations with ONE patient/relatives*’
S3 satisfaction ‘perceived appreciation by the team’ bad to very bad (4/202)	
Other professions decide on the need for psychological support (*n* = 2)	‘*Colleagues often want us to go to patients who don't want to talk to us at all, which I firmly reject. Sometimes “less” is more. Some other practitioners – often nursing staff – think they know better what patients need from us than we psychologists do as professionals. They assume too much that we act like a pill that can be used to wipe away all problems from one day to the next shortly before the patient's end of life*’
Too little exchange within and with the multiprofessional team (*n* = 2)	‘*There is only a weekly exchange about the patients*’
Palliative psychological support is not considered important by the multiprofessional team (*n* = 1)	‘*The need for a psychologist is often not recognised*’
Lack of time/personnel resources (*n* = 1)	‘. . . *there is simply not enough time for the patients*’
Personal conflicts with leader (*n* = 1)	‘*Personal conflicts with the palliative care physician in charge*’
S4 satisfaction with ‘supervision, intervision and exchange in the multiprofessional team’ bad to very bad (18/201)	
Lack of supervision/intervision (*n* = 14)	‘*There is none in the palliative care team that I could take part in*’
Lack of time or structural resources (*n* = 7)	‘*And as there is already far too little time, exchanges and intervision are undesirable in the work*’ ‘*There are hardly any resources available for this, neither my time (within working hours, for example) nor financial resources provided by the clinic*’
A lack of collegial exchange (*n* = 4)	‘*Collegial exchange not sufficient*’
Too many different responsibilities (*n* = 4)	‘*I am only called in for individual consultations, participation in team meetings or similar is not remunerated*’ ‘*We psycho-oncologists have our own supervision, which is great. In palliative supervision, we also often tend to take on the role of helpers*’
Lack of supervision quality (*n* = 3)	‘. . . *we could bring in cases in our POS supervision, but the supervisor has little experience with palliative situations*’
S5 ‘overall satisfaction’ bad to very bad (5/204)	
Dissatisfaction with the team (*n* = 2)	‘*The expectations of the multiprofessional team are often very high, you can never get it right. In meetings, you often find yourself “alone against everyone” with your opinion, you can’t do enough. . .*’
Lack of understanding the roles and tasks of palliative psychology (*n* = 2)	‘*Other practitioners also don’t seem to realise that patients have the right to refuse offered therapies/interventions. They are often far too weak to do so!*’ ‘*No interdisciplinary treatment plan. . .*’
Poor communication and unfavourable leadership behaviour (*n* = 1)	‘*Poor communication and poor management behaviour on the part of the doctors responsible*’
Lack of time/personnel/structural resources (*n* = 1)	‘. . . *too little time for several consultations with ONE patient, no own office*’

## Discussion

This cross-sectional survey aimed to make palliative psychological care in Germany more transparent and to obtain an insight into self-assessed satisfaction facets with the work as well as the professional attitudes of the psychologists/psycho-oncologists surveyed.

### Discussion of the descriptive results in relation to palliative psychological care in Germany

The results show that palliative psychological care varies greatly depending on the type of palliative care facility. Psychologists/psycho-oncologists appear to work most frequently in palliative care units and least frequently in inpatient hospices. The low number of psychologists/psycho-oncologists in the outpatient sector reflects the fact that in Germany only the professional group of doctors and nurses is currently financed by the health insurance funds.^56^ This is consistent with the findings of the report on psycho-oncological care in Germany, where in outpatient hospice and palliative care, psychological/psycho-oncological support was mainly provided by physicians, social workers or palliative care nurses, most of whom did not have any further psychological/psycho-oncological training.^28^ Especially in the home setting, the burdens experienced by patients and relatives are likely to require and justify palliative psychological expertise to a particular degree.^57–60^ The lack of funding for the third professional group for specialist palliative home care has already been emphasised very critically by the DGP and brought to the attention of politicians as a matter that cannot be postponed.^61^ Even if these figures cannot be interpreted as an exact image of palliative psychological care in Germany, a trend can be deduced that has also been observed in other countries. A survey of hospices in the United Kingdom showed, that despite established guidance on structuring psychological support in palliative care for adults, and although psychological care in the hospice sector has improved over the last 15 years, it is far from being perceived as adequate by staff.^62^ Not only the prompt availability of trained psychologists for more complex symptom burden needs to be improved, but also the level of expertise in terms of mental health symptom assessment, counselling and communication among (non-psychological) professionals.^62,63^ In an Australian survey of palliative care facilities for adults (PCOC – Palliative Care Outcomes Collaboration), access to psychological or psychiatric expertise was perceived as very unsatisfactory.^64^ Respondents reported that most psychological support was provided by social workers (94.1%), spiritual care workers (62.5%) or creative therapists (43.8%). Psychiatrists or psychologists only provided this type of support in 31.3% and 25%, respectively. A large proportion (60%) stated that they had no access to psychological or psychiatric experts. Here, too, the inadequate systematic assessment of psychological distress was noted.^64^

The weekly working hours of the psychologists/psycho-oncologists surveyed varied between the institutions and were also lowest in hospices. It is striking that the average working time in the POS was considerably lower than in the 2018 report on psycho-oncological care (where it was given as *M* = 19.3, SD = 11.2 for the inpatient area).^28^ This may be because the participants in this study were asked to divide the hours into ‘palliative’ and ‘less palliative’. Over half of the respondents stated being firmly integrated into the MPCT, although only about a third stated working exclusively in a purely palliative setting. This suggests that team integration does not necessarily depend on whether someone works exclusively in a MPCT.

The fact that only half of the respondents worked predominantly with palliative patients, many of the respondents had an additional psycho-oncological qualification and only a small proportion had completed further training in palliative care for psychologists indicates that palliative psychological care in Germany is predominantly provided by psycho-oncologists. This is also supported by the comments from the free text fields (see Table 10), in which it was frequently described that there are agreed and changing responsibilities for palliative patients in addition to psycho-oncological work. This was reported above all when several colleagues were responsible for the palliative area. Another aspect that supports this trend is the result that only about three quarters have completed a psychology degree at master’s level. In Germany, qualification as a psycho-oncologist does not necessarily require a degree in psychology, unlike specialist psychologists in palliative care.^33,65^ The extent to which these differences in qualifications affect the nature and quality of psychological work or influence the outcome for patients and their relatives has not yet been investigated. More than half stated (at least) one licence to practise as a psychological psychotherapist, which roughly matches the qualification profile of psycho-oncologists in the survey of psycho-oncological care in Germany in 2018.^28^ The existence of further support services corresponds in part to the figures from a Germany-wide survey of palliative care units on the structure and care of patients, whereby it was not investigated how exactly palliative psychological care was integrated and provided (unpublished results).^66^ Despite the recommendations for organising MPCTs,^2^ very little is known nationally^10^ and internationally^67^ about the actual professionals involved in the various sectors. This applies not least to the psychological professional group.^9^ The study by Gahr et al.^68^ revealed major differences in the multiprofessional staffing of palliative care support teams in German hospitals within Comprehensive Cancer Centres (CCC). Psychologists/psycho-oncologists were only represented in 7 of 13 palliative care support teams. This should be seen critically, as early integration of palliative psychological expertise increases treatment quality more likely for both patients and their relatives, especially in the case of non-oncological diseases.^69^ In terms of the implementation of best practice recommendations (e.g. MPCT composition) for high-quality palliative care within CCCs, the structural quality appears to be very heterogeneous across Germany to date.^70,71^

### Discussion of the results on professional attitude and satisfaction facets

Overall, the respondents rated their professional attitude as recommended in the specialist literature and described as helpful for the needs of the seriously ill, the dying and their relatives.^3,18,72^ An open-ended, unintentional, interested, not knowing attitude that creates presence for fear-free spaces; an attitude that considers not only expertise but also intuition; an attitude that makes it possible to actively address difficult topics, such as the presence of death wishes. Nevertheless, a higher level of agreement (after recoding) was expected for statement ‘enter contact with a clear mission’. In the focus group discussion, the attitude ‘I find it important for my work with the seriously ill, the dying and their relatives to enter into the first contact with a clear mission’ was rated as a very unfavourable palliative psychological attitude. For this reason, the item was expected to be answered negatively. Strada emphasises this aspect in particular when she recommends ‘to maintain awareness of personal cultural beliefs, attitudes and biases that may impact care of patients from different cultural backgrounds’ (p. 179) and encourages to ‘regard the patient and the family as the “experts” on their culture and their personal circumstances, and demonstrate willingness to be educated and informed by them about what they consider valuable’ (p. 179).^3^ It is not uncommon for psychologists/psycho-oncologists that to ask team members to support patients in coming to terms with their illness.^69^ This often means, for example, accepting a change in treatment goals, recognising that death is approaching or accepting palliative care treatment/interventions for better symptom control. Aspects such as existential distress^58^ or wishes to hasten death^73^ can play a role here, which can themselves lead to high levels of stress in the MPCT.^74^ Such a request from the team is understandable, because they experience the patient or the environment as very burdened by a lack of acceptance or compliance. From the patient’s perspective, however, it may be an appropriate and suitable coping strategy for their needs. To accept the team’s mandate uncritically would disregard the patient’s construction of reality and values. The fact that other professional groups (mostly physicians) decide on the need for psychological support (and formulate this as an order) was also cited as a reason for dissatisfaction in the free text responses. This is an aspect that is also criticised by Oberth et al.^69^ There, the authors emphasise that it is essential for psychologists/psycho-oncologists to get a picture of the patient/relatives themselves and assess their symptom burden and support needs. As non-psychological professionals in the healthcare sector are not necessarily able to correctly classify psychological distress, they often see the suffering as a problem to be solved and may arrive at different assessments than psychologists or psycho-oncologists. This item was presented inversely and a tendency to agree potentially influenced the response behaviour.^75^ It is also conceivable that this is due to socialisation as a therapist/psycho-oncologist in the healthcare sector, where the desire to ‘fix’ is widespread among the vast majority of helping professions.^76,77^ Furthermore, the difficulty in rejecting the team’s mandates for fear of being perceived as ‘uncooperative’ or understanding them as a mandate to act.

Overall, satisfaction with integration into and appreciation by the MPCT, perceived effectiveness, supervision, intervision and exchange as well as overall job satisfaction appear to be good to very good among the respondents in this survey. The satisfaction facets all correlate positively (in some cases very highly) with each other, which suggests that they contain similar or even complementary facets of the overarching construct of job satisfaction.^78^ This would also be supported by the good internal consistency of Cronbach’s alpha (*α* = 0.82) when the five items are combined into one scale.^79^ A strikingly high correlation between S1 and S3 indicates that satisfaction with the integration into and the appreciation experienced by the MPCT are related. Such a correlation was also found in another sample, where it also positively influenced the feeling of team identity.^80^ The significant correlations between age and S2 and PE and S2 are supported by statements from clinical psychologists who reported needing at least 6 months to a year to familiarise themselves with working in the palliative care field.^4^

### Discussion of the results of the investigation of group differences

Based on the assumption that the quantity of work with the seriously ill, dying and their relatives makes a difference in terms of professional attitude and satisfaction facets, the participants were assigned to a predominantly palliative and a less palliative setting. In this sample, there were no significant differences in the attitudes of the psychologists/psycho-oncologists between the groups. This may have been due to the fact that, overall, few participants were located in an exclusively palliative setting, or only a few had completed further training in palliative care for psychologists. It is also conceivable that psychologists/psycho-oncologists, regardless of whether they work mainly with cancer patients in a curative setting or primarily with the dying in a palliative setting, have a similar self-image of their role and approach people with the same kind of attitude. Such an attitude also corresponds to the core aspects for professionals in palliative care described by Simon et al.^81^

Also, psychologists/psycho-oncologists who worked predominantly in a palliative setting appeared to be more satisfied with their integration into the MPCT, their perceived effectiveness and their overall job satisfaction. Even if the effect sizes in this sample are only small to medium, this can potentially be interpreted as a positive trend. These differences have not yet been explicitly investigated, but studies with psycho-oncologists and palliative psychologists indicate that, in addition to common aspects of satisfaction, there are definitely different stress factors that influence the experience of satisfaction.^35,36^

The few participants who felt bad to very bad integrated into the MPCT in this study most frequently justified this with a lack of time and personnel resources, too many responsibilities in different areas and a lack of dialogue with team colleagues. This may reflect the effects of how palliative psychological care is currently structured in Germany. It is not unusual for the multiprofessional approach in palliative care to yield different perspectives and treatment suggestions from team members.^82^ To ensure that this multiperspectivity is not a potential source of conflict but a resource for staff and, not least, for patients and their relatives, regular dialogue is essential. MPCT meetings provide the necessary space to negotiate the best possible patient-centred care with each other.^83^ In addition, this type of regular communication also contributes to the prevention of burnout and compassion fatigue.^40,41^

When respondents in this study rated their effectiveness as bad to very bad, this was also due to insufficient time and resources for the individual patient. In Oberth et al. psychologists/psycho-oncologists from a palliative care support team within a hospital tended to rate their effectiveness generally only in the medium range (measured as the psychological benefit of their work).^69^ The reasons given for this were difficulties in establishing contact due to psychological defences, high need for autonomy, high symptom burden or reservations about psychological support services. Similar obstacles were not reported by the participants in this study. If overall satisfaction was rated as bad to very bad and reasons for this were given, this was most likely due to team conflicts, unfavourable communication or leadership, or the impression that the role of palliative psychology in the team is not really understood or valued. These results once again emphasise the importance of a well-developed team culture^82,83^ and the central role of a team leader,^84^ who should shape precisely these processes. At the same time, this may also stem from the heterogeneity of qualifications and the fact that only a small proportion had undergone further training in palliative care for psychologists. Even though the overall level of satisfaction with supervision, intervision and exchange in this sample was high and no difference was apparent with regard to the setting, attention should nevertheless be paid to the comments of those who gave reasons for their dissatisfaction in this regard.

In this sample, the level of professional experience had no significant influence on the respondents’ professional attitude. This is surprising, as it could be assumed that this type of attitude only emerges with increasing experience and the development of a certain degree of self-reflection in dealing with death and dying.^4,85^ However, this could also be an indication that this area is mainly staffed by psychologists/psycho-oncologists who already bring along a high level of professional experience that is already linked to an attitude that is described as supportive and helpful in dealing with seriously ill and dying patients and their relatives. With regard to the ‘effectiveness’ satisfaction facet, there were differences between those with little compared to those with medium and high professional experience. Participants who have worked with the seriously ill, the dying and their relatives for more than 3 years perceived themselves as more effective than those with less experience in the palliative field. This is in line with the findings of the study by Fan et al.^4^ and emphasises the necessity of giving oneself time to find a way to respond to the needs of those affected in a helpful way in this particular field of work. This development process involves not only time and self-reflection, but also the use of supervision, intervision and continuous further education and training.^8,25,86^

### Strengths and limitations

To our knowledge, this is the first Germany-wide study of psychologists/psycho-oncologists in palliative settings for adults that has examined structural conditions, professional attitudes and satisfaction measures. Even the recruitment of the targeted sample proved to be more challenging than expected. Of the 1072 palliative care facilities contacted, less than a third were able or willing to establish further contact with the responsible psychologist/psycho-oncologist. It remains unclear whether the remaining facilities did not forward the invitation to the study to their responsible psychologist/psycho-oncologist, whether there were no responsible psychologists/psycho-oncologists there or whether the invitation was forwarded but the addressees themselves failed or were unwilling to make contact/participate. Due to the voluntary nature of participation exists a selection bias, which limits the generalizability of the results. For this reason, the results can only be classified as a trend and cannot provide a representative picture of palliative psychological care in Germany.

Less than half of those who received a personalised link to the study actually completed the questionnaire. In view of the fact that 58 links were either not functional or did not reach the participants, the question arises as to whether other technical obstacles led to questionnaires not being processed. Besides obstacles, a lack of interest or inattention, it may also be that the announced total processing time (for the complete survey) of 30–35 min seemed too long for many. Future studies could take this aspect into account and favour shorter surveys.

Since this study was of exploratory nature, we did not use validated scales containing a set of items for measuring the professional attitude or the extent of satisfaction. As a result, the presented items showed little discriminatory power, and the distribution was striking left skewed. This may also have contributed to the fact that only a few group differences and low effect sizes were found. This weakness could be addressed in the future by using validated scales for the constructs of interest. Since it was not clear before the development of the questionnaire that so many participants were working in different settings at the same time, it was challenging to determine a predominantly and less palliative setting based on the data. It may well be that the allocation made does not optimally reflect the actual distribution. This distinction should definitely be made more precisely in a future study. Another point that weakens the significance of the mean value comparisons is the artificial dichotomisation^87,88^ of the setting and the use of a categorised^89^ variable for professional experience. This procedure was chosen due to the fact that the target ordinal-scaled variables analysed were not normally distributed and a regression analysis procedure would have been inappropriate.^90^

Despite these weaknesses, it was possible to include psychologists/psycho-oncologists from all palliative settings in the study and to obtain an approximate idea of how psychological care is structured in Germany. On a positive note, the participation rate of 85.5% and completion rate of 87.1% can be considered high, confirming the findings of Suppan et al., who showed that recruitment with personalised links is more successful than providing a general access link.^91^ However, the participation rate cannot be reliably compared with other studies in the healthcare sector, as the reporting of participants is often not correctly oriented towards the CHERRIES guidelines.^92^ It can be seen as a success that the professional attitude of psychologists/psycho-oncologists has become so clearly visible for the first time. The contents of training and further education of future palliative psychologists can build on these results, integrate them and develop and specify additional aspects. Finally, the high satisfaction ratings can be seen as an indication that, despite all the challenges, the work with the seriously ill, the dying and their relatives and carers is still a very rewarding experience, which confirms the results of Cramond et al.^36^ To date, psychologists/psycho-oncologists in the palliative setting have hardly been studied with regard to their job satisfaction or related effects such as burnout, emotional exhaustion or compassion fatigue.^38^ A review showed that applied psychologists in different work areas are susceptible to burnout, especially emotional exhaustion, due to their high emotional and cognitive engagement and responsiveness with their patients.^93^ Due to the special requirements in the work with palliative patients and their relatives, this professional group should also become the focus of researchers. It should not be overlooked that reported dissatisfaction in this study was often caused by a lack of dialogue within the multiprofessional team, too many responsibilities, unclear role requirements or insufficient time resources. This can be valuable information for responsible stakeholders and leadership to initiate structural improvements.

## Conclusion

The findings of this study indicate that patients are most likely to receive palliative psychological support during care on a palliative care unit. In view of the psychological distress experienced by patients and relatives, for example, after a previous change in treatment goal, this can certainly be interpreted as a reasonable and positive result. At the same time, the question arises as to why there are currently so few psychologists/psycho-oncologists in palliative care support teams within hospitals or inpatient hospices as well as in the specialist palliative home care. Such structural heterogeneity appears unsatisfactory not only for patients and their relatives in terms of successful multiprofessional palliative care, but also for those who have to fill such a gap as a team member without being sufficiently equipped to do so. If professional treatment and support on the four dimensions are to be successful in all palliative settings, then these results should be taken as an opportunity to rethink the current care structures. Recording the associated professions in specialist palliative care facilities could be a valuable first step towards making the structures more transparent. More homogeneous palliative psychology expertise could be specifically promoted by making further training to become a certified specialist psychologist for palliative care a prerequisite for working in this field.
